# Vectra 3D (dinotefuran, pyriproxyfen and permethrin) prevents acquisition of *Borrelia burgdorferi* sensu stricto by *Ixodes ricinus* and *Ixodes scapularis* ticks in an ex vivo feeding model

**DOI:** 10.1186/s13071-021-04881-5

**Published:** 2021-08-21

**Authors:** Djamel Tahir, Btissam Asri, Leon Nicolaas Meyer, Alec Evans, Thomas Mather, Byron Blagburn, Reinhard K. Straubinger, Valérie Choumet, Frans Jongejan, Marie Varloud

**Affiliations:** 1 Clinvet Morocco, B.P 301, 28815 Mohammedia, Morocco; 2 Institut Agronomique Et Vétérinaire Hassan II, Rabat, Morocco; 3 Center for Vector-Borne Disease, University of Rhode Island, Kingston, RI USA; 4 College of Veterinary Medicine, Auburn University, Auburn, AL 36849 USA; 5 Institute for Infectious Diseases and Zoonoses, Bacteriology and Mycology, Faculty of Veterinary Medicine, Ludwig-Maximilians-University Munich, 80539 Munich, Germany; 6 Environnement Et Risques Infectieux, Institut Pasteur, Paris, France; 7 Vectors and Vector-Borne Diseases Research Programme, Department of Veterinary Tropical Diseases, Faculty of Veterinary Science, University of Pretoria, Onderstepoort, South Africa; 8 Ceva Santé Animale, 10 Avenue de la Ballastière, 33500 Libourne, France

**Keywords:** Canine Lyme borreliosis, *Ixodes scapularis*, *Ixodes ricinus*, *Borrelia burgdorferi*, Blocking transmission, Prevention, Ex vivo model

## Abstract

**Background:**

We evaluated the efficiency of an ex vivo feeding technique using a silicone membrane-based feeding chamber to (i) assess the anti-feeding and acaricidal efficacy of a spot-on combination of dinotefuran, pyriproxyfen and permethrin (DPP, Vectra® 3D) against adult *Ixodes scapularis* and *Ixodes ricinus* ticks, and to (ii) explore its effect on blocking the acquisition of *Borrelia burgdorferi* sensu stricto.

**Methods:**

Eight purpose-bred dogs were randomly allocated to two equal-size groups based on body weight assessed on day 2. DPP was administered topically, as spot-on, to four dogs on day 0. Hair from the eight dogs was collected individually by brushing the whole body on days 2, 7, 14, 21, 28 and 35. On each day of hair collection, 0.05 g of sampled hair was applied on the membrane corresponding to each feeding unit (FU). Seventy-two FU were each seeded with 30 adults of *I. scapularis* (*n* = 24 FU) or *I. ricinus* ticks (*n* = 48 FU). Bovine blood spiked with *B.* *burgdorferi* sensu stricto (strain B31) was added into each unit and changed every 12 h for 4 days. Tick mortality was assessed 1 h after seeding. One additional hour of incubation was added for live/moribund specimens and reassessed for viability. All remaining live/moribund ticks were left in the feeders and tick engorgement status was recorded at 96 h after seeding, and the uptake of *B. burgdorferi* s.s. was examined in the collected ticks by applying quantitative real-time PCR.

**Results:**

Exposure to DPP-treated hair was 100% effective in blocking *B. burgdorferi* s.s. acquisition. The anti-feeding efficacy remained stable (100%) against both *Ixodes* species throughout the study. The acaricidal efficacy of DPP evaluated at 1 and 2 h after exposure was 100% throughout the study for *I. ricinus*, except the 1-h assessment on day 28 (95.9%) and day 35 (95.3%). The 1-h assessment of acaricidal efficacy was 100% at all time points for *I. scapularis*.

**Conclusions:**

The ex vivo feeding system developed here demonstrated a protective effect of DPP against the acquisition of *B. burgdorferi* without exposing the animals to the vectors or to the pathogen.

**Graphical Abstract:**

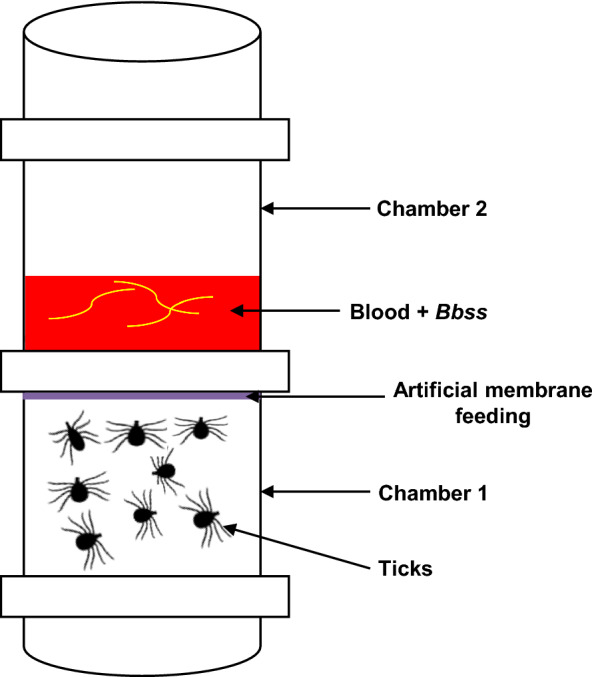

## Background

Ticks are obligate hematophagous ectoparasites that parasitize vertebrate animals (birds, mammals and reptiles) and occasionally attach to humans [1]. Within the Ixodidae family (also called hard ticks), *Ixodes* is the largest genus, containing 217 species [2]. Ixodid ticks transmit a larger variety of infectious agents, including viruses, bacteria (rickettsiae and spirochaetes), protozoa and helminths, than any other arthropod vector group, and are among the most important vectors of infections affecting livestock, companion animals and humans [1, 3, 4].

Ticks in the genus *Ixodes* are most familiar as vectors of the Lyme disease (LD) spirochete (*Borrelia burgdorferi*). *Ixodes ricinus*, known as the sheep tick or castor bean tick, is the most abundant European tick species but it is also widespread in North Africa [5, 6]. On dogs, this tick species can act as a vector of many pathogens including *Borrelia burgdorferi* sensu lato, *Anaplasma phagocytophilum* and *Babesia* spp., which are the causative agents of Lyme borreliosis (LB), canine granulocytic anaplasmosis and babesiosis, respectively [6]. Within the North American *Ixodes* species, the blacklegged tick, *Ixodes scapularis*, is the most frequently incriminated vector for *B. burgdorferi* sensu stricto and *A. phagocytophilum* in dogs [7]. All stages of *I. ricinus* and *I. scapularis* can feed on dogs and bite humans, enabling the transmission of several pathogens to people including those causing LB, rickettsiosis, anaplasmosis, babesiosis, tick-borne encephalitis, Powassan virus disease and many more [8, 9]. Immature *I. scapularis*, at least, also can acquire an infectious dose of the LD agent while feeding on dogs [10].

Preventing tick bites and thus the transmission of tick-borne pathogens requires the use of molecules that target the tick and/or pathogen survival [11]. Protecting dogs from tick infestation is commonly achieved by periodic application of effective repellent and acaricidal agents. Nevertheless, the relevance of these chemical compounds in blocking pathogen transmission is related to the speed of transmission of the specific pathogen by its tick vector [12]. This time of transmission may vary between pathogens and is influenced by numerous variables. Some studies have shown that transmission can occur much faster than expected [13, 14]. Therefore, pharmaceutical compounds should be characterized by a fast onset of killing activity and/or repellence against arthropods [12].

Although vaccination offers an alternative approach for preventing tick-borne pathogen infections, currently the protective spectrum of vaccines is limited, and there are none available for most of the canine tick-borne diseases [15, 16].

Recently, several artificial feeding methods of ixodid ticks have been developed to study tick-host–pathogen interactions under laboratory conditions [17–22]. Artificial membrane feeding devices were successfully used to evaluate the killing efficacy of chemicals or biogenic compounds such as acaricides and antimicrobials used against hematophagous arthropods. For instance, Kröber and Guerin [23] demonstrated the possibility of using membrane feeding to evaluate the acaricidal effect of fipronil and ivermectin against female *I. ricinus* ticks feeding through a silicone membrane. Similarly, the effectiveness of ivermectin and doxycycline in killing body lice was assessed using a parafilm membrane and human blood as an in vitro system [24].

In this study, we investigated an ex vivo feeding model, wherein hair collected from dogs previously treated with Vectra 3D (dinotefuran, pyriproxyfen and permethrin) was introduced to assess its ability to prevent the acquisition of *B. burgdorferi* s.s. by *I. ricinus* and *I. scapularis* ticks.

## Methods

### Dog study design

**Fig. 1:**
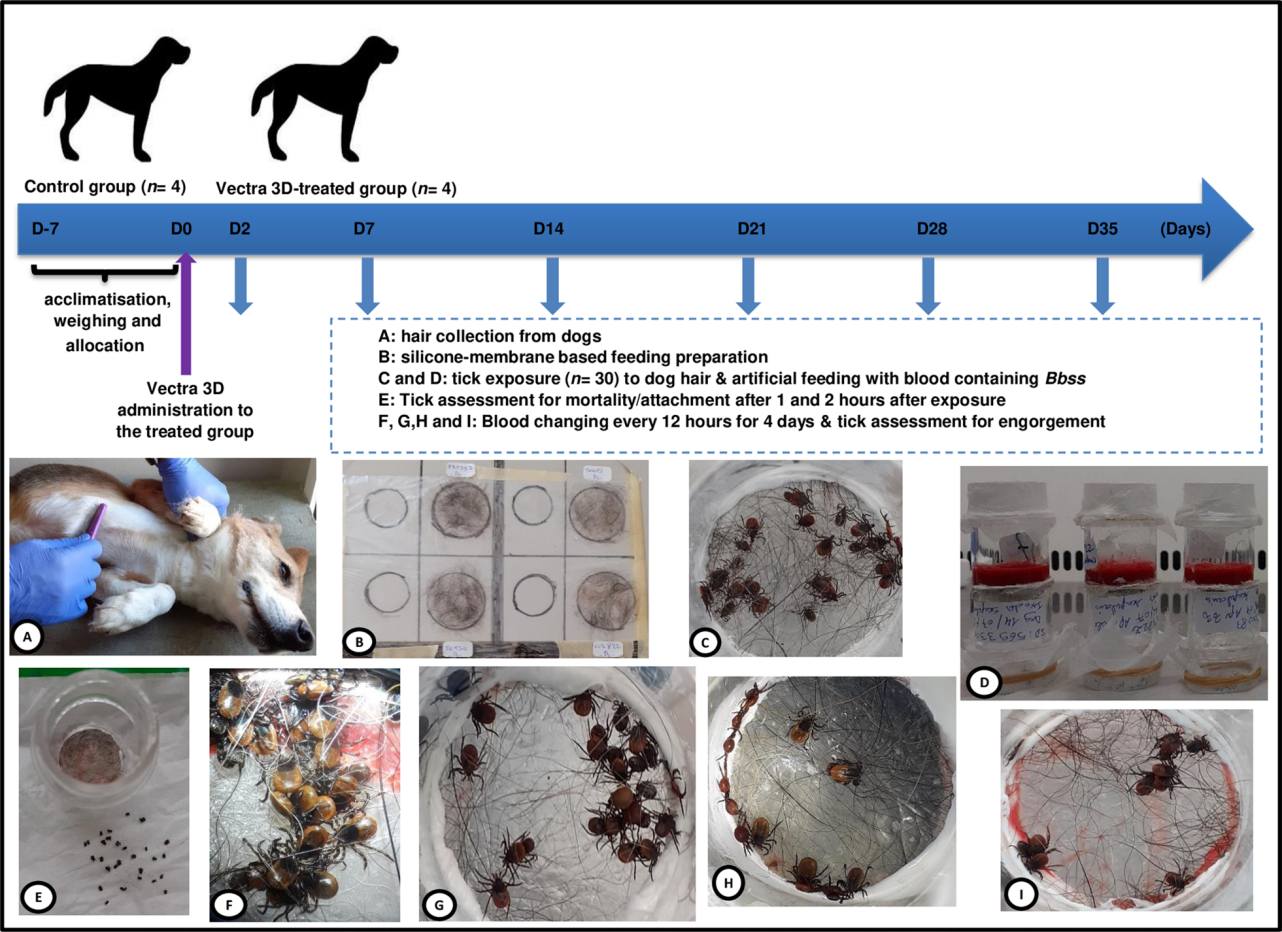
Summary of the experiments conducted in this study. **a** Hair collection on a dog using an individual comb. **b** Preparation of silicone-based membrane. **c** Chamber 1 filled with 30 ticks. **d** Dead ticks assessed after 1 h of DPP-treated dog hair contact. **e** *I. ricinus* clustering on the artificial membrane 3 days after tick infestation. **f** Feeding units made from two plexiglass chambers, chambers 1 and 2, containing ticks and blood, respectively. **g** Feeding unit illustration: chamber 1 with organza tissue at the base to restrain ticks; chamber 2 containing blood with end covered with sterile parafilm; artificial membrane glued between chambers 1 and 2. **h** Cluster of semi-engorged adult *I. scapularis* ticks feeding on the artificial membrane. **i**, **j** Partially engorged *I. scapularis* ticks after 84 h of feeding

Eight healthy purpose-bred Beagle dogs were clinically examined on day 7 for inclusion in the study. On day 2, the dogs were randomly assigned to two groups (dinotefuran-permethrin-pyriproxyfen [DPP] or untreated control group) of four dogs each according to their body weight. The dogs were individually housed indoors at a temperature of 24 °C (±3 °C). No physical contact was allowed between animals from the different groups. The dogs were fed a commercial dry food at the recommended rates and water ad libitum. General health observations were performed once daily for the duration of the study. On day 0, DPP was administered topically based on the manufacturer’s instructions [25] as a line-on treatment along the length of the spine to four dogs (1.6 ml/dog weighing between 5 and 10 kg and 3.6 ml/dog weighing between 10 and 25 kg). Hair (about 3.2 ± 1.8 g/dog) from the eight dogs was collected from each individual by brushing the whole body (using single-use brushes for DPP-treated dogs) over a period of about 10 min, on days 2, 7, 14, 21, 28 and 35 (Fig. 1).

### Ticks

Adult female and male *I. ricinus* and *I. scapularis* (at the age of 6 to 8 weeks after molting) were obtained from Clinvet’s colony initiated in 2012 using adult specimens from Georgia (USA) and Utrecht (the Netherlands), respectively, collected in the field by F. Jongejan. Pathogen-free ticks were maintained by routine passage on rabbits for larvae and nymphs, and on sheep for adult ticks. Prior to use in the study, ticks were maintained at 10–12 °C and relative humidity (RH) of 85–95% (Panasonic Corporation, Osaka, Japan). To increase their willingness to feed, ticks were transferred to 22 °C, 90% RH and 16:8 h light/dark photoperiod at least 5 days prior to tick feeding challenges.

### Bacterial strain

The *B. burgdorferi* sensu stricto strain B31 (*Bb*ss; ATCC 35210; American Type Culture Collection, Manassas, VA 20110 USA) was used in this experiment. *Borrelia* cultures were maintained in complete Barbour-Stoenner-Kelly medium (BSK-H) at 34 °C as described previously [26]. The spirochete cell viability and concentration were determined by counting spirochetes in defined suspension volumes using a dark-field microscope.

### Membrane and tick feeding chamber preparation

The feeding unit (FU) design was prepared based on a method described previously [23, 27]. Briefly, the feeding units were made by two plexiglass tubing chambers stacked on each other (Fig. 1). Chamber 1 (containing ticks) was separated from chamber 2 (containing blood) by a previously prepared silicone-based membrane which was gently fixed to the feeding chamber using mastic silicone glue (SikaSeal®, Ballymun, Ireland). Feeding membranes consisted of goldbeater’s skin originally made from bovine intestine (Preservation Equipment Ltd, Norfolk, UK) with a thickness of 30 μm, which were treated with a thin layer of silicone rubber (Smooth-On, Inc., East Texas, PA) mixture to improve the softness, resulting in a final membrane thickness of 100–140 µm. The silicone mixture was prepared as reported previously [27]. Just after adding the silicone mixture, 0.05 g of dog hair (DPP-treated or non-treated control) was added to the membrane in the circle corresponding to the placement of the feeding chamber (Fig. 1). The membrane was allowed to polymerize overnight at room temperature.

### Preparation of blood seeded with *Bb*ss

Blood was collected weekly from *Borrelia*-free cattle (Clinvet's facility) in 500 ml sterile collection bags supplemented with citrate (MacoPharma, Mouvaux, France). The blood was supplemented with glucose (Sigma-Aldrich, St. Louis, MO, USA) to a concentration of 2 g/l and stored at 4 °C. Prior to blood replacement, the required volume of blood was warmed to 37 °C. Blood was then spiked with BSK-H medium containing spirochetes to obtain a final concentration of 10^4^ *Bb*ss cells/ml. The blood was changed every 12 h for 4 days, and during this process, all chambers containing blood (chamber 2) were rinsed at least twice with warm 0.9% NaCl containing gentamicin (10 mg/ml; Sigma-Aldrich) and then left to dry for 5 min under a biosafety cabinet.

### Tick seeding, incubation, mortality and engorgement assessment

Each FU was filled with 30 adult ticks (1:1 sex ratio) of *I. ricinus* on days 2, 7, 14, 21, 28 and 35 or *I. scapularis* on days 2, 14 and 28. The availability of *I. scapularis* did not allow for testing at every time point. Three milliliters of prepared bovine blood containing *Bb*ss was added into each FU and then sealed with sterile parafilm (Fig. 1).

FU were incubated at 37 °C with humidity > 85%. Tick mortality was assessed 1 h after exposure to the hair. However, one additional hour of incubation was added to determine the survival of the live and/or moribund specimens. The engorgement status of all remaining live ticks was assessed after 4 days of incubation. Ticks were categorized with the naked eye as semi-engorged or non-engorged. However, a stereomicroscope was used to detect traces of a blood meal when differentiation was not possible with the naked eye.

It should be noted that wherever possible, the guidelines for evaluating the efficacy of parasiticides in reducing vector-borne pathogen transmission, recently published by the World Association for the Advancement of Veterinary Parasitology (WAAVP) were properly followed [29].

### DNA isolation and qPCR screening of the ticks

After each collection time point, ticks were washed twice in phosphate-buffered saline (PBS, Sigma-Aldrich) and dried. Ticks were then labeled and stored at −20 °C until DNA extraction. DNA was extracted from whole ticks using a NucleoSpin tissue kit according to the manufacturer’s recommendations (Macherey–Nagel, Hoerdt, France). For the non-treated group, DNA was extracted from all semi-engorged females, while for the treated group, three specimens per feeder per time point were randomly selected and DNA was individually extracted. In addition, nine attached males classified as semi-engorged were tested for *Bb*ss. The uptake of *Bb*ss organisms was examined by quantitative real-time polymerase chain reaction (qPCR), as described previously [30]. Individual ticks were processed separately, and spirochete acquisition was considered successful when the number of threshold cycles (Ct) was < 36. Negative controls were processed with DNA-free water and DNA from uninfected ticks, and the positive control was genomic DNA of *Bb*ss.

### Efficacy assessment and statistical analysis

Live tick counts (attached or free and engorged or non-engorged) were transformed to the natural logarithm of (count + 1) to calculate the geometric means (GM) at each time point for both *Ixodes* tick species. The efficacy was calculated using Abbott's formula (Abbott 1987):

${\text{Acaricidal efficacy }}\left( {\text{\% }} \right) = 100{\text{ x }}\frac{{{\text{MC}} - {\text{MT}}}}{{{\text{MC}}}}$, where MC is the GM of live ticks in the control group (group 1), and MT is the GM of live ticks in the treated group.

${\text{Antifeeding efficacy}} \left( \% \right) = 100 \times \frac{{{\text{MC}} - {\text{MT}}}}{{{\text{MC}}}}$, where MC is the GM of engorged ticks in the control group and MT is the GM of engorged ticks in the treated group.

The percentage effectiveness of DPP in preventing *Bb*ss acquisition by ticks was calculated as follows:

${\text{Efficacy}} \left( \% \right) = 100 \times \frac{{{\text{PC}} - {\text{PT}}}}{{{\text{PC}}}}$, where PC is the percentage of ticks positive for *Bb*ss based on qPCR in the untreated control group and PT is the percentage of positive ticks in the treated group.

Data were analyzed using Excel to calculate the GM and standard deviation. Statistical analyses were performed using GraphPad Prism 6 software (https://www.graphpad.com/). At each time point, differences between treated and untreated groups were compared using the non-parametric two-tailed Mann–Whitney *U* test. Pearson’s *χ*^2^ test was performed for analysis of the engorgement rates between the two species, and Fisher’s exact test was used to evaluate the differences in the prevalence of spirochete acquisition. Values of *p* < 0.05 were considered statistically significant.

## Results

All treated dogs tolerated the DPP administration, with no abnormal signs occurring throughout the study. In the non-treated group, both tick species exhibited normal behavior when exposed to dog hair in the feeding chamber, and tick attachment was observed during the first 2 h of release into the chamber. There was no tick mortality recorded in this group for either species during the first hours of assessment (1 and 2 h of incubation), yielding a GM of 30 live ticks (Table 1). At each subsequent challenge, some non-attached ticks were found dead, particularly between 2 and 4 days of incubation, but this mortality did not exceed 12% (Table 1).

**Table 1:** Mortality assessment of adult *Ixodes ricinus* and *Ixodes scapularis* ticks exposed for 1 and 2 h to dog hair treated with dinotefuran-permethrin-pyriproxyfen or not treated

Day of exposure	*Ixodes ricinus*						*Ixodes scapularis*		
	1 h			2 h			1 h		
	Non-treated control	DPP-treated group		Non-treated control	DPP-treated group		Non-treated control	DPP-treated group	
		Dead tick count (%)	Mean tick count ± SD		Dead tick count (%)	Mean tick count		Dead tick count (%)	Mean tick count
2	0	120 (100)	30 ± 0	0	120 (100)	30	0	120 (100)	30
7	0	120 (100)	30 ± 0	0	120 (100)	30	N/A	N/A	N/A
14	0	120 (100)	30 ± 0	0	120 (100)	30	0	120 (100)	30
21	0	120 (100)	30 ± 0	0	120 (100)	30	N/A	N/A	N/A
28	0	114 (95.0)	28.5 ± 1.9	0	120 (100)	30	0	120 (100)	30
35	0	113 (94.1)	28.2 ± 1.2	0	120 (100)	30	N/A	N/A	N/A

*SD* standard deviation, / no experiment conducted for *Ixodes scapularis* due to limited tick availability

**Fig. 2:**
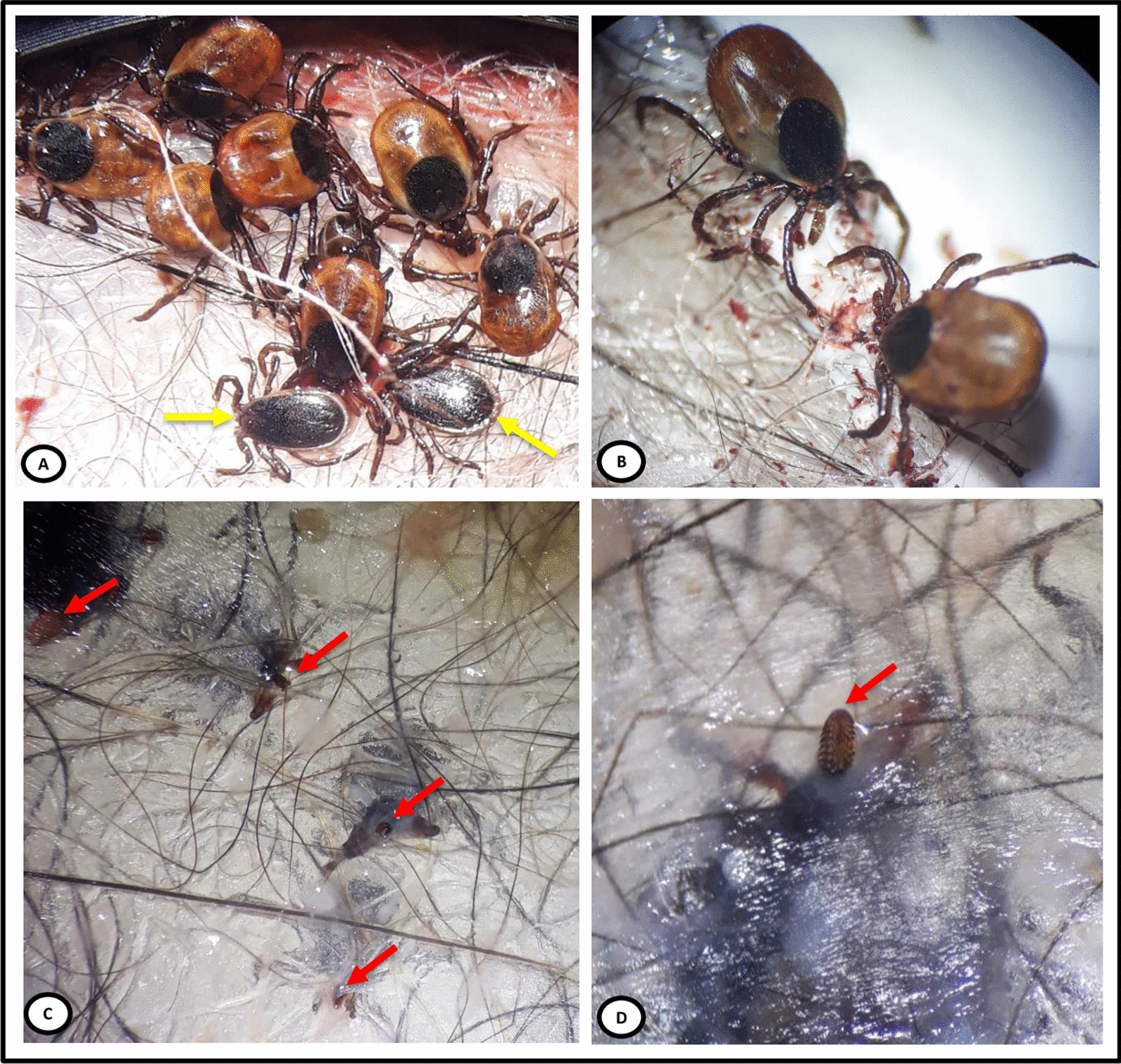
Engorgement status of adult *Ixodes* ticks on the silicone membrane following 4 days of tick feeding. **a** Group of partially engorged *I. scapularis* females; example of males considered attached and semi-engorged are indicated with yellow arrows. **b** Semi-engorged females *I. ricinus.* **c**, **d** Outside view of the adult tick hypostomes perforating the silicone membrane, indicated with red arrows

In the untreated control group, ticks successfully attached and fed through the artificial feeding membrane, with engorgement values (recorded after 96 h of incubation) ranging from 20.0 to 36.7% (27.7 ± 1.9%) for *I.* *ricinus* and from 36.6 to 41.6% (39.4 ± 1.9%) for *I. scapularis* (Table 3). Female *I. scapularis* fed better than female *I. ricinus*, with a statistically significant difference (*X*^*2*^ = 7.54, *p* = 0.006). We occasionally observed attached and slightly engorged males (Fig. 2), with a total of nine males categorized as semi-engorged. The GM of semi-engorged ticks ranged from 3.3 to 6.4 and from 6.7 to 7.2 for *I. ricinus* and *I. scapularis*, respectively (Table 2). It should be noted that only female ticks were considered in this assessment (each feeder contained 15 females) (Table 3). *Bb*ss DNA was detected in a total of 96% (96/100) and 95.7% (68/71) of partially engorged female *I. ricinus* and *I. scapularis*, respectively (Table 4). A total of eight partially engorged males (*I. scapularis*: *n* = 6/7 and *I. ricinus*: *n* = 2/2) were tested positive for *B. burgdorferi*.

**Table 2:** Anti-feeding efficacy of DPP-treated dog hair against *Ixodes ricinus* and *Ixodes scapularis* ticks

Day of exposure	*Ixodes ricinus*^a^				*Ixodes scapularis*^a^			
	Total number of fed ticks (%) in non-treated control groups	GM number of fed ticks ± SD		Anti-feeding efficacy (%)	Total number of fed ticks (%) in non-treated control groups	GM number of fed ticks ± SD		Anti-feeding efficacy (%)
		Non-treated control groups	DPP-treated groups			Non-treated control groups	DPP-treated groups	
2	22 (36.7)	6.4 ± 1.3	0	100	24 (40.3)	6.7 ± 2.4	0	100
7	17 (28.3)	5.2 ± 0.9	0	100		N/A	N/A	N/A
14	18 (30.0)	5.4 ± 1.3	0	100	22 (36.7)	5.9 ± 3.1	0	100
21	15 (25.0)	4.7 ± 0.9	0	100		N/A	N/A	N/A
28	12 (20.0)	3.3 ± 2.1	0	100	25 (41.6)	7.2 ± 1.0	0	100
35	16 (26.7)	4.9 ± 0.8	0	100		N/A	N/A	N/A

^a^Only females were considered during the assessment. Each feeding chamber contained 15 female ticks

*GM* geometric mean, *SD* standard deviation, / = no experiment conducted for *Ixodes scapularis* due to limited tick availability

**Table 3:** Acaricidal efficacy of DPP-treated dog hair against *Ixodes ricinus* and *Ixodes scapularis* ticks

Day of exposure	*Ixodes ricinus*						*Ixodes scapularis*		
	GM number of alive ticks				Acaricidal efficacy (%)		GM number of alive ticks		Acaricidal efficacy (%)
	1 h		2 h		1 h	2 h	1 h		1 h
	Non-treated controls	DPP-treated group	Non-treated controls	DPP-treated group			Non-treated controls	DPP-treated group	
2	30	0	30	0	100	100	30	0	100
7	30	0	30	0	100	100	N/A	N/A	N/A
14	30	0	30	0	100	100	30	0	100
21	30	0	30	0	100	100	N/A	N/A	N/A
28	30	0.97	30	0	96.9	100	30	0	100
35	30	1.45	30	0	95.3	100	N/A	N/A	N/A

/ no experiment conducted for *Ixodes scapularis* due to limited tick availability

**Table 4:** Preventive effect provided by DPP-treated dog hair against the acquisition of *Bb*ss by *Ixodes ricinus* and *Ixodes scapularis* ticks

Day of exposure	*Ixodes ricinus*^a^			*Ixodes scapularis*^a^		
	Total number of tested/positive ticks (%)		Efficacy (%)	Total number of tested/positive ticks (%)		Efficacy (%)
	Non-treated control groups	DPP-treated groups		Non-treated control groups	DPP-treated groups	
2	22/22 (100)	0/12 (0)	100	21/24 (87.5)	0/12 (0)	100
7	17/17 (100)	0/12 (0)	100	N/A	N/A	N/A
14	18/18 (100)	0/12 (0)	100	22/22 (100)	0/12 (0)	100
21	14/15 (93.3)	0/12 (0)	100	N/A	N/A	N/A
28	10/12 (83.3)	0/12 (0)	100	25/25 (100)	0/12 (0)	100
35	15/16 (93.7)	0/12 (0)	100	N/A	N/A	N/A

^a^Only females were considered during the assessment, / no experiment conducted for *Ixodes scapularis* due to limited tick availability

In the DPP-treatment group, the ticks exhibited signs of intoxication, such as agitation, then uncoordinated movements and “hot-foot”-like behavior after only 15 min of contact with the DPP-treated hair. The proportion of dead *I. ricinus* assessed after 1 h of incubation was 100% (GM = 30) during the first four challenges (days 2, 7, 14 and 21), which decreased slightly to 95% (GM = 28.5) and 94% (GM = 28.2) on days 28 and 35, respectively (Table 1). The 2-h mortality for this tick species was 100% for each of the time points (Table 1). For *I. scapularis*, 100% (GM = 30) mortality was noted at each time point during the first 1 h of incubation (Table 1). The immediate 1-h acaricidal efficacy of DPP-treated hair against *I. ricinus* ticks remained above 95% and reached 100% at the 2-h counts (Table 3). This efficacy was 100% at 1 h for *I. scapularis* at each time point (Table 3). For both tick species, there was a significant difference between the treated and control groups (*U* test, *p* value varied between 0.006 and 0.01) in the number of dead ticks found at 1 and 2 h of incubation.

No attachment was observed in the DPP-treated group. DPP had 100% anti-feeding effect against both tick species throughout the study (Table 2). At each time point, the difference in the engorgement mean of female *I. ricinus* and *I. scapularis* between the treated and control groups was significant (*p* < 0.001).

All of the randomly selected female ticks from the DPP-treated group (*I. ricinus*: *n* = 72 and *I. scapularis*: *n* = 36) tested negative for *B. burgdorferi* DNA (Table 4). Thus, the preventive effect provided by DPP-treated dog hair against the acquisition of *Bb*ss by *I. ricinus* and *I. scapularis* was 100% throughout the study period (35 and 28 days for *I. ricinus* and *I. scapularis*, respectively). For both tick species, there was a significant difference in the expected proportion of positive ticks between the control and the DPP-treated groups on Fisher's exact test (*p* < 0.00001).

## Discussion

The ex vivo membrane-based feeding system developed here was successfully adapted to test the efficacy of Vectra 3D treatment of dog hair in preventing *B. burgdorferi* acquisition by adult *I. ricinus* and *I. scapularis* ticks over a period of 4 weeks. The 100% blocking efficacy reached at each time point was attributable to both the anti-feeding efficacy and the fast acaricidal effect against ticks observed during the first hours of exposure to treated hairs.

Efficacy assessment of veterinary products with acaricidal properties is usually performed on the target animal (e.g. cattle, cats, dogs, rabbits). Indeed, in experiments focusing on the transmission of vector-borne diseases, the animals are exposed not only to the bites of the vectors, but also to the infection by the pathogens they transmit [31, 32]. In vivo procedures are stressful and painful for animals due to often massive tick bite burden as well as infection if the ticks are infected. In the case of LB studies, the pre-patent period required to rule out previous infection and the holding time, typically 4–6 weeks after infection, before serology can turn positive means that animals are on study for a lengthy time period. The goal of the 3R principles (replace, reduce and refine) is to always replace animal experiments whenever possible, reduce the number of animal experiments to the lowest possible, and ensure that the distress inflicted upon the animals is as low as possible. Alternative and more rapid methods for achieving the same study objectives while also reducing animal exposure and manipulation are thus desirable. In this study, we demonstrated the utility of an ex vivo artificial blood feeding assay system for assessing the efficacy of a quick tick knockdown product in preventing tick biting and feeding but where more than 80% of the activities previously performed on the dog, such as sedation, tick infestation and tick counts, were avoided.

In the current ex vivo assay, application of dog hair on the membrane as a chemical and mechanical *stimulus* appears to be an important factor in producing a satisfactory rate of tick attachment and engorgement (median engorgement rate of 27.8 and 39.4% for *I. ricinus* and *I. scapularis*, respectively) after only 4 days of tick incubation. In previous studies, bovine hair, sheep wool and deer hair extracts were applied to membranes to enhance attachment of immature and adult ixodid ticks fed in vitro [21, 28, 33]. In addition to the chemical stimulus, Kröber and Guerin [23] placed plastic crosses (2-mm-thick tile spacer) on the membrane to create additional borders where ticks prefer to attach. In nature, attachment of ticks at feeding sites on the host also depends on an appropriate array of chemical and physical stimuli [34], where hair likely plays a key role.

In the present study, the number of ticks attached on the membranes was not recorded at each blood replacement, because it was not actually feasible to categorize them as attached or not without checking them individually using tweezers, which can disrupt the tick attachment process. In their study, Kröber and Guerin reported a 77% attachment rate by female *I. ricinus*, but only 54% of these females were still alive and feeding after 9 days of incubation. By comparison, the engorgement rate for *I. ricinus* reported here was lower (27.7%), and this could be due to several parameters including the shorter period (4 days) ticks were allowed to feed and considering the possibility that some ticks may attach at a later time. When infestation is performed on target animals, the WAAVP recommend leaving ticks on the animals until completely engorged. As for *I. scapularis*, the median engorgement rate obtained in the present study was 39.4%, which was close (33–50%) to the frequency reported by Oliver et al. [35] when ticks were given 7 days of access to feed on an artificial membrane. Despite experiencing the same experimental conditions, the engorgement rate for *I. scapularis* (39.4%) was significantly greater (*p* = 0.006) than that for *I. ricinus* (27.7%). We observed that *I. scapularis* ticks appeared more active than *I.* *ricinus*, which may explain the difference in the engorgement rate.

Even though it is usually considered that adult male ticks of the genus *Ixodes* do not require a blood meal for spermiogenesis [36], in this study a number of male *I. ricinus* and *I. scapularis* males were occasionally observed to be attached directly on the membrane. When their engorgement status was assessed with a stereomicroscope, a total of 9/540 specimens were classified as semi-engorged. To the best of our knowledge, only one study involving artificial feeding of *Ixodes* spp. ticks has reported the occurrence of blood feeding by males; in that study, 11% of feeders containing only *I. ricinus* males were found with the presence of feces inside [37]. In the present study, detection of *B. burgdorferi* DNA in eight male ticks suggests that adult *Ixodes* males may be capable of acquiring this bacterium during blood feeding. This hypothesis deserves to be investigated by both in vitro and in vivo studies using male *Ixodes* spp. ticks. Male *Rhipicephalus sanguineus* sensu lato, *Dermacentor andersoni* and *Dermacentor reticulatus* can take blood feeding on the host, and can both acquire and transmit pathogens in the absence of female ticks [13, 37–39].

Following a topical application of Vectra 3D, the three active substances (dinotefuran, permethrin and pyriproxyfen) were shown to be rapidly distributed over the body surface of the animal within the first day, with maximum concentrations on the hairs obtained 3 days after the application [26]. All three active substances were still detected in different zones of the hair coat 1 month after treatment [25]. The anti-feeding and acaricidal efficacy of DPP against adult *I. ricinus* [40] and *I. scapularis* [41] was already shown to persist on dogs for 1 month. The 24-h mortality assessed ex vivo against nymphal *I. scapularis* was 100% at all time points except on days 10 (97.9%) and 17 (96.5%) [42]. Interestingly, this formulation demonstrated remarkable speed of kill of both *Ixodes* tick species tested here. We know of no other product used for dogs as an acaricide that claims 100% effectiveness in just 1 to 2 h of tick contact with treated hair/animal for the entire month. This speed of kill action against *I. ricinus* and *I. scapularis* ticks is explained by the presence of permethrin (36.08%). Permethrin is known to be a fast-acting acaricide; it acts after contact with the arthropod and absorption into the arthropod either directly through the outer cuticle or through ingestion during feeding on the host [43, 44]. The lipophilic properties of permethrin promote its distribution along the arthropod nervous system. Permethrin, like all pyrethroids, acts as a neurotoxin on voltage-gated sodium channels by slowing their activation and inactivation properties, leading to hyperexcitability and death [45]. In addition to its acaricidal effects, permethrin exerts a potent repellent and knockdown effect against numerous arthropods including ticks, and the primary repellent activity of pyrethroids is via contact irritancy [44]. This property is a key factor for the prevention of tick attachment on the host, which could explain why in the feeders containing DPP-treated hair, none of *I. ricinus* and *I. scapularis* scored positive.

In this study, DPP completely prevented acquisition of *B. burgdorferi* by *I. ricinus* and *I. scapularis* ticks at each time point throughout the 1-month period following product administration. While not tested here, we might expect similar results if the experiment had used infected ticks and uninfected blood, since the transmission blocking effect is obtained through anti-feeding and acaricidal action on the vectors. *Ixodes ricinus* and *I. scapularis* ticks are mainly incriminated in the transmission of *B. burgdorferi* s.l., agent of LD. During a blood meal on the host, infected ticks release a small number of microorganisms into the skin. In general, inoculation of an infectious dose of bacteria by the tick bite occurs after 24–48 h [46]. However, for certain *Borrelia* strains (e.g., *Bb*ss strain BRE 13), it has been observed that the transmission may occur within the first 12 h [14]. Therefore, to be effective in preventing transmission of tick-borne pathogens to pets, the most effective preventive products should prevent blood-feeding.

## Conclusions

This study demonstrated a successful blocking effect of DPP against transmission of *Borrelia* bacteria, using an ex vivo artificial blood feeding chamber. A 100% acaricidal effect was achieved within 1–2 h after *I. ricinus* and *I. scapularis* ticks were in contact with DPP-treated hair, preventing tick attachment and engorgement. A single administration of DPP to dogs blocked transmission of *Borrelia* for up to 5 weeks due to strong anti-feeding and acaricidal efficacy against ticks.

## Data Availability

Data supporting the conclusions of this article are included within the article and its additional file. Further data of interest will be available from the corresponding author upon request.

## Abbreviations

*Bb*ss: *Borrelia burgdorferi* sensu stricto
*Bb*sl: *Borrelia burgdorferi* sensu lato
LB: Lyme borreliosis
FU: Feeding units
DPP: Dinotefuran, pyriproxyfen and permethrin
GM: Geometric mean
RH: Relative humidity
